# Sustainable Materials: A Conceptual Gap in Definition

**DOI:** 10.3390/ma19101957

**Published:** 2026-05-09

**Authors:** Mohammad Reza Saeb

**Affiliations:** Department of Pharmaceutical Chemistry, Medical University of Gdańsk, J. Hallera 107, 80-416 Gdańsk, Poland; mr.saeb@gumed.edu.pl

**Keywords:** sustainable materials, sustainability metrics, lifecycle, materials selection, materials classification, circular economy

## Abstract

The term “sustainable material” has become widely used in materials science, yet its conceptual foundation remains inconsistently interpreted. Despite major progress in sustainability assessment, circularity metrics, life cycle analysis, and policy-oriented frameworks, sustainability is still commonly treated as an intrinsic material property instead of a multidimensional and context-dependent outcome. This perspective addresses this unresolved conceptual gap by critically distinguishing widely used but often interchangeably applied terms such as bio-based, recyclable, biodegradable, circular, green, renewable, and sustainable materials. It is argued that none of these descriptors alone can define sustainable materials, since each captures a specific or only a limited aspect of material behavior while overlooking interacting factors, including processing conditions, infrastructure, embodied impacts, end-of-life management, and application-dependent constraints. Accordingly, the present work challenges binary classification of materials as “sustainable” or “non-sustainable” and proposes a shift toward a more integrated and context- dependent interpretation of sustainable materials. By clarifying the boundaries and limitations of existing terminology, this perspective aims to strengthen scientific communication, improve sustainability-oriented materials selection, and motivate the development of more structured multidimensional frameworks for future “sustainable material” classification.

Materials science has witnessed significant progress across major material classes, including metals and alloys, polymers, ceramics and glasses, as well as natural materials such as wood and stone, through advances in synthesis and processing strategies such as green chemistry–assisted synthesis [1], controlled polymerization processes [2], innovations in metallurgical design and alloy engineering [3], the development of advanced blends and composites [4], and functional glass systems [5], supported in part by developing computational [6] and manufacturing [7] capabilities. However, such progress does not necessarily guarantee full alignment with environmental and resource-related constraints that continuously shape materials systems [8]. In this regard, “sustainable material” have evolved as a conceptual class of materials directing attention toward reducing environmental burdens, enabling circular use of resources, and supporting the transition toward low-carbon material systems [9]. This perception reflects not merely technological advancement, but also the growing recognition that the performance of material must be interpreted beyond functionality alone, in relation to lifecycle, resource efficiency, and broader environmental impact. Nevertheless, despite this recognition, the concept of sustainability in materials science—and, in particular, the term “sustainable material”—remains implicit in definition and selectively interpreted; thus, it is often known and treated as an intrinsic characteristic rather than as a multidimensional and context-dependent explicit outcome.

The lack of conceptual clarity has already been well documented, as pioneering foundational work on sustainability terminology has shown that the propagation of related terms, their inconsistent use, and the lack of clear relationships among them can significantly hinder technological communication [10]. The terminology surrounding sustainability and sustainable materials becomes more heterogeneous when considering recent advances in materials science, along with challenges in sustainable consumption and production [11]. In spite of progressive developments in the field of materials science and technology, such as materials for bio-integrated devices [12], energy storage materials [13], electromagnetic interference (EMI) shielding materials [14], self-healing materials [15], lightweight composite materials [16], and metamaterials [17], several fundamental questions regarding sustainability and sustainable materials remain limitedly addressed, particularly with respect to how sustainability is defined, interpreted, and consistently applied across different material systems and circumstances. At the same time, the term “sustainable material” has been increasingly used across research [18], industry [19], and decision-making [20] bodies, while its meaning remains only partially aligned across different contexts. Nevertheless, over the past two decades, this field has advanced markedly through the development of sustainability indicators, circularity-oriented approaches, reporting frameworks, and policy-driven standards, even though such progress has not fully resolved the conceptual inconsistency surrounding the term “sustainable material” [21]. This unresolved inconsistency provides the point of departure for the present perspective.

The focus of this work is on demonstrating this conceptual gap and clarifying why sustainability cannot be interpreted explicitly as an intrinsic material property, but rather as a context-dependent outcome arising from multiple interacting factors, which in turn motivates the main questions addressed in this perspective. The primary contribution of the present work lies in communicating this conceptual gap and showing how the current use of sustainability-related terminology may lead to scattered and possibly deceptive interpretations in materials science. In particular, the present perspective aims to clarify how sustainability-related terminology is used across different contexts, why such usage leads to conceptual inconsistencies, and how this affects both scientific communication and practical decision-making in materials selection. Therefore, it addresses a central paradox of the field: although methods, indicators, reporting systems, and governance frameworks have become significantly more sophisticated, the underlying terminology remains insufficiently integrated with regard to the term “sustainable material”. This limitation in defining “sustainable material” gives rise to a set of essential questions that must be addressed before sustainability can be meaningfully interpreted and consistently applied in materials selection:

- What does a sustainable label mean, actually, when applied to a material?
- Can sustainability be defined based on a single property, such as recyclability or renewable origin?
- Why are terms such as green, bio-based, recyclable, and sustainable often used interchangeably?
- Is it meaningful and practically viable to classify materials simply as “sustainable” or “non-sustainable”?

Together, these questions shift the discussion from terminology alone toward the way sustainability affects materials selection, evaluation, and communication, thereby linking conceptual ambiguity with its practical implications and guiding the subsequent analysis. These questions extend beyond conceptual considerations and directly influence how sustainability is understood in materials science, potentially leading to practical concerns and misunderstandings. However, depending on how sustainability is defined, it directly influences the standards and metrics by which materials are designed, selected, evaluated, and communicated. This is particularly important today, as sustainability-related evaluation increasingly interacts with indicator-based systems, reporting architectures, and policy-oriented frameworks that require conceptual clarity in order to remain scientifically meaningful and operationally consistent [22,23]. When fundamental definitions, widely used by researchers and engineers, are unclear and applied paradoxically, decisions based on these definitions must frequently be revised and may become largely unreliable and unpredictable [24]. For example, materials science has traditionally focused on understanding and optimizing properties such as strength, durability, conductivity, and stability. Therefore, materials are conventionally selected based on their ability to meet functional requirements and function in a given application, highlighting the need for both strategy and tools for materials selection [25,26]. For example, metals are generally chosen for structural performance and load-bearing capacity, polymers for flexibility and lightweight applications, ceramics and glass for thermal and chemical resistance, and natural materials such as wood and paper for availability and ease of processing. Each class of conventional materials brings specific advantages, but also inherent limitations. However, these conventional considerations alone become insufficient once sustainability is introduced as an additional dimension in materials selection. Therefore, the shift from conventional materials selection toward sustainability-oriented decision-making has gradually introduced broader scientific, methodological, and institutional expectations that extend beyond the performance of materials alone [27].

Extending this discussion to advanced materials, and with increasing attention and emphasis directed toward sustainability, an additional layer of evaluation has been introduced [28]. Materials are no longer evaluated solely based on performance, but increasingly on their environmental consequences. In contrast to conventional material descriptors, sustainability cannot be meaningfully represented through a single measurable parameter, but instead develops from the interplay of multiple considerations. A central misconception addressed in this work lies in the oversimplified interpretation of sustainability, where it is often approached as a permanent characteristic, despite its inherently multi-factor and context-dependent nature. In fact, sustainability cannot be associated with a specific characterization technique or a single representative variable. Instead, it is often inferred indirectly, based on selected characteristics that are assumed to be environmentally favorable. This indirect and selective interpretation contributes significantly to the divergence and fragmentation of definitions observed across the literature. Often, sustainability is associated with a particular feature of a material. For example, a material derived from biomass is described as bio-based and is often identified as sustainable because it originates from renewable resources. Likewise, a material that can be reprocessed is sometimes labeled as recyclable and is considered environmentally beneficial because it can enter several more production cycles. In a similar manner, a material that degrades under certain conditions is sometimes described as biodegradable and is assumed to have minimal long-term environmental burden. Although these associations and labels are useful, they remain incomplete independently [29,30].

When used as independent indicators of sustainability, these terms may lead to oversimplified conclusions and reinforce the misconception that sustainability can be attributed to a single defining feature. This limitation becomes even more pronounced when processing conditions and system-level factors are taken into account. Processing routes, energy intensity, and technological requirements may significantly alter the environmental footprint of a material, even when its intrinsic properties appear favorable [31,32]. Accordingly, sustainability should be understood as an outcome shaped by the combined influence of material characteristics and process-dependent factors, rather than as a singular defining feature [33]. This points to a broader conceptual issue, in which sustainability is often approached through static interpretations, rather than being recognized as an outcome dependent on the interaction between material characteristics and the conditions under which they are produced, used, and managed at the end of life. From a practical standpoint, the sustainability of materials depends on multiple interacting factors rather than separated characteristics. In addition to material characteristics, these factors include processing routes, production intensity, energy demand, and associated embodied impacts such as cumulative energy consumption and CO_2_ emissions, which may significantly influence the overall sustainability profile of materials in practice [34].

A bio-based material may require severe agricultural contributions or energy-intensive processing. Likewise, a recyclable material may not necessarily be recycled in practice due to economic or infrastructural limitations. This further indicates that practical implementation, including collection systems, technological feasibility, and infrastructure availability, plays a pivotal role in determining whether sustainability-related potential is realized [35]. Accordingly, a biodegradable material may require controlled conditions that are not commonly available. In each of these examples, a favorable characteristic is of interest, but it does not absolutely determine the sustainability of the material. An additional layer of complexity is fueled by the coexistence of different terminologies that are often used interchangeably. Terms such as green, eco-friendly, sustainable, renewable, recyclable, and circular appear frequently in the literature, yet they are often applied without clear distinction despite not having identical definitions. Some of these terms are descriptive, others are regulatory, and some others are used primarily for communication rather than for scientific precision. Thus, it is possible that the same material may be labeled differently depending on the intention of the author and/or decision-making considerations. This situation leads to divergent definitions and interpretations of sustainability, where instead of a unified concept, it becomes a collection of weakly connected perspectives, which further complicates its application under real system-level conditions. Addressing this disintegration may require more systematic approaches in the future, including quantitative analyses of terminology usage across the literature, identification of dominant research communities and application domains, and synchronized efforts toward more structured and operational definitions.

From an environmental perspective, one study may emphasize carbon emissions; another may focus on resource origin, while a third may consider the end-of-life behavior of materials. Accordingly, life cycle assessment (LCA) has emerged as one of the most widely accepted and scientifically viable methodologies [36], yet it does not resolve the underlying conceptual ambiguity addressed in this work. Although LCA provides a powerful framework for environmental assessment, it does not by itself constitute a universally accepted conceptual definition of what makes a material “sustainable”, as its outcomes depend on methodological choices such as system boundaries, assumptions, and selected impact categories [37]. Although each perspective is valid, their lack of integration makes it difficult to compare outcomes or to draw general conclusions. This lack of integration represents one of the central elements of the conceptual gap addressed in the present work. In this sense, the coexistence of multiple methodologies—such as circularity-oriented indices and LCA—reflects both the progress of the field and the underlying conceptual fragmentation that motivates the present study. Therefore, achieving greater consistency in defining sustainable materials may ultimately depend on collective efforts within the scientific community, where insights from different domains are systematically integrated rather than treated separately.

Another particularly important problem in defining “sustainable material” is the lack of a basis for distinction between potential and actual performance. Many sustainability-related terms used in the literature describe what a material could make under the assumption of ideal or specific conditions, where some solutions were proposed in terms of metric of sustainability (MOS) [38]. From materials science perspective, however, more dimensions should be principally considered. For instance, a material may be technically recyclable, but its recyclability remain mostly theoretical if it is not collected, sorted, and processed. Likewise, a compostable material may demand industrial capacities that are not generally available. In such cases, the assumed environmental benefit is not realized in practice. In addition to these conceptual challenges, there is also a general tendency among players in the field of materials science to simplify sustainability into a binary classification, where materials are often labeled as either sustainable or non-sustainable, based on selective standards. However, this approach does not necessarily capture the real picture of material systems. In fact, most materials exhibit both favorable and unfavorable characteristics. For example, a metal may be durable and recyclable but require intensive production energy. Likewise, a polymer may be lightweight and economically favorable, yet difficult to process or manage at the end of its life. Most critically, a natural material may be renewable but limited in durability or performance. Thus, both the definition and classification of sustainable materials remain selective, fragmented, and conceptually incomplete, reflecting the absence of a unified framework for interpreting sustainability across material systems.

Narrowing the practical complexity of defining “sustainable material” into a binary label not merely oversimplifies the problem but may also lead to misleading conclusions. In fact, the intention of this study is not to disregard or question the term “sustainable material”, but to highlight that its current usage often indicates a universally valid property that may not adequately reflect the context-dependent nature of sustainability. In other words, the current term used worldwide may lead to the wrong assumption that sustainability is an inherent property of a material. Nevertheless, sustainability should additionally address how the material is processed, produced, applied, and managed, as these factors directly influence both scientific communication and practical decision-making. Therefore, prior to defining or classifying sustainable materials, it is necessary to investigate how sustainability-related terms are currently used and what they actually mean [39]. The present work does not aim to provide a definitive solution, but rather to highlight the conceptual gap that currently limits consistent interpretation and application of sustainability in materials science. Correspondingly, this perspective aims to provide a clearer basis for understanding why current terminology remains insufficient, and how this limitation affects both scientific communication and decision-making in materials selection and interpretation. A logical reconsideration of these terms helps to identify their limitations, avoid misinterpretation, and support more consistent approaches in the future. In particular, it becomes essential to distinguish how these terms are applied in different contexts, which of them are most frequently used in practice, and how their interpretation may vary depending on material class, application, and system-level conditions and considerations. This provides the rationale for summarizing the most common sustainability-related descriptors before discussing their broader implications.

In light of the above discussion, Table 1 presents a conceptually simplified overview of commonly used terms associated with sustainable materials, serving as a structured basis for comparing their scope and limitations. In particular, it illustrates how the current use of terminology contributes to fragmented interpretations of sustainability across material systems, thereby supporting the central argument of this work. The table demonstrates how these terms are applied across metals, polymers, natural materials, and composites, emphasizing the potential for misinterpretation when used individually. It is important to note that the table summarizes widely used and often overlapping descriptors, in order to highlight how these terms, although useful individually, are frequently used interchangeably despite representing fundamentally different aspects of material behavior. It should also be emphasized that the table does not aim to define “sustainable material” directly, but instead serves to clarify what each term captures, what it neglects, and how it is often misunderstood. By classifying these terms in a disciplined manner and linking them to representative material examples across different classes, this table highlights the diversity of interpretations alongside existing gaps in current terminology in the domain of materials science.

The overview presented in Table 1 highlights an essential observation and suggests that commonly used terms in the framework of sustainable materials are not principally equivalent, and they also do not necessarily capture the same aspects of material behavior. It should also be emphasized that the interpretation and relevance of these terms may vary or be revised in the future depending on application scenarios, system boundaries, available infrastructure, processing conditions, energy consumption, carbon emission, and policy-attributed considerations, which independently or simultaneously further underline the need for context-related evaluation rather than uniform classification. Table 1 should be regarded as a conceptual illustration of how terminological assumptions determine the success or failure of sustainability-oriented interpretations, particularly when terms with different definitions are used interchangeably as “sustainable material” indicators. Accordingly, it becomes evident that each term should be used with careful consideration of its specific scope, rather than being applied broadly as a general indicator of “sustainable material”. This table simply reveals that each term reflects a specific picture, focused on a single characteristic, whereas other relevant aspects remain unaddressed. From this perspective, it becomes clear that these terms cannot be used interchangeably, nor can they be merged to form so-called novel terms as a complete definition of sustainable materials. As such, a material may be biodegradable but not necessarily or completely recyclable; or it can be recyclable without showing low-carbon features when applied in practice, illustrating that these descriptors reflect different dimensions rather than equivalent indicators of “sustainable material”.

Terms related to resource origin, such as renewable and bio-based, emphasize the source of materials and the need to reduce dependence on finite resources, yet do not principally provide information about how materials are processed, how they behave during service, or how they behave after their service life. Likewise, a bio-based material may still bring about significant environmental consequences in the processing course, or may not degrade effectively under real conditions. Likewise, terms related to end-of-life behavior, such as recyclable, biodegradable, and compostable, focus on what happens to a material after its service life. These terms are often interpreted optimistically, as recognized within the field, suggesting that materials can be recovered or safely degraded, but their practical relevance depends strongly on external factors such as infrastructure, collection systems, and environmental conditions. A recyclable material is not necessarily recycled, and a biodegradable material does not always degrade in uncontrolled environments. Terms such as low-carbon or circular also endeavor to capture broader aspects of sustainability. However, even these terms are often applied selectively. A material with low carbon emissions may still involve trade-offs in other areas, such as resource use or toxicity. Likewise, the concept of circularity depends not merely on material properties but also on the systems that enable reuse and recycling [40]. In recent years, organized efforts have been made to quantify such circularity-related aspects through reliable indices and evaluation frameworks, particularly focusing on end-of-life performance and material recovery pathways, which represent important progress toward operationalizing sustainability-related characteristics [41]. However, such approaches typically address specific stages or dimensions of material life cycles; therefore, they do not fully capture upstream impacts, processing burdens, or broader system-level interactions that contribute to the overall sustainability profile of materials.

The inclusion of example materials in Table 1 further illustrates how these terms are applied across different material classes. Metals such as aluminum and steel are often associated with recyclability and circularity, polymers such as polylactic acid (PLA) are associated with bio-based origin and biodegradability, and natural materials such as wood and paper are closely linked with renewability and degradability. As reflected in Table 1, none of these materials can be adequately described through a single descriptor. By contrast, each term exhibits a combination of favorable and limiting characteristics that must be considered simultaneously. This observation reinforces the idea that “sustainable material” cannot be defined through a single characteristic. It suggests that any future attempt to classify or define “sustainable material” must account for multiple interacting dimensions in a structured and context-assisted manner, originating from the interaction of multiple factors. Focusing on a single characteristic may lead to incomplete or even misleading conclusions, particularly when materials are compared across different applications or systems. It is also believed that the limitations of current terminology should highlight the need to move beyond binary classification, treating materials simply as “sustainable” or “non-sustainable”.

In light of the above clarifications, instead of asking whether a material is sustainable, it becomes more meaningful to ask how sustainability is expressed and which factors contribute most significantly to its overall profile, reflecting a shift from binary classification toward a more context-aware interpretation. This standpoint supports a more comprehensive understanding of “sustainable material” and strengthens materials selection and evaluation processes. At the same time, it is important to acknowledge that sustainability is not determined solely by intrinsic material properties. External factors such as processing methods, application conditions, and infrastructure play a crucial role in determining how materials perform in practice. A material that appears favorable in view of sustainability under laboratory conditions may behave differently at larger scales in industrial systems. These considerations suggest that defining “sustainable material” is challenging and requires more than a collection of favorable attributes. This also demands a coordinated and comprehensive approach that takes into account multiple aspects of material behavior and their interactions. Although Table 1 does not explicitly provide a complete definition, it serves as an essential step in clarifying the boundaries of commonly used terms and identifying the gaps that must be addressed. It can be interpreted not as a classification scheme, but as a conceptual basis for recognizing the limitations of current terminology and motivating the development of more structured approaches in the future.

Looking ahead, it is believed that several challenges remain in advancing the understanding of the definition and classification of sustainable materials:

- How can sustainability be defined in a way that provides both a structured and adaptable framework across different material classes?
- How can carbon emissions be evaluated alongside resource use, performance, and end-of-life behavior without oversimplification?
- How can infrastructure and technological differences be systematically integrated into sustainability assessment?
- How can developing materials be evaluated consistently under standard conditions when data is incomplete or uncertain?

Addressing these challenges in relation to “sustainable material” definition requires a paradigm shift from individual and descriptive use of terminology in materials science toward more disciplined and multidimensional approaches.

In the broader framework of sustainability science and practice, it is important to recognize that substantial efforts have already been made over the past decades to structure, quantify, and operationalize sustainability through various frameworks, standards, and institutional initiatives [42]. International organizations and policy-driven platforms, including those associated with the United Nations [43], the European Union [44], and other global and regional bodies, have contributed significantly to shaping sustainability-related principles, reporting structures, and evaluation methodologies [45]. More recent developments—including the EU Green Deal [46], the Corporate Sustainability Reporting Directive (CSRD) [47], the European Sustainability Reporting Standards (ESRS) [48], and the Business Responsibility and Sustainability Report (BRSR) [49]—have significantly expanded this landscape, yet they operate within a terminology that remains conceptually unresolved. These advancements clearly demonstrate that the field has matured considerably in terms of tools, metrics, and institutional support. However, despite this progress, a fundamental limitation remains; i.e., these efforts are often developed across partially independent domains and are not necessarily integrated into a unified conceptual framework specific to materials science. As a result, while sustainability is increasingly operationalized through guidelines, indicators, and reporting systems, its interpretation at the level of materials—particularly in terms of a clear and universally accepted definition of “sustainable material”—remains implicit. This observation highlights that the challenge is not the absence of activity or progress, but rather the lack of conceptual integration across existing knowledge systems.

From this perspective, it becomes increasingly evident that future progress in defining and classifying “sustainable material” will require a more integrated and multidimensional approach, consistent with the conceptual gap identified throughout this work. In such a framework, sustainability is best understood as an outcome emerging from the interaction of multiple factors operating across different levels. These include material-related attributes such as resource origin, processing routes, performance during use, and end-of-life behavior, together with system-level considerations such as energy demand, carbon emissions (including CO_2_-related impacts), infrastructure availability, and policy frameworks that govern practical implementation. In this context, materials selection for “sustainable material” framework may be viewed as a layered decision-making process, where different dimensions—including environmental indicators such as embodied energy and emissions—must be considered simultaneously rather than separately. Although the present work does not aim to define a definitive framework or classification scheme, it highlights the necessity of moving toward more structured and integrative approaches in the future. In particular, it suggests that global efforts involving systematic analysis of terminology, engagement with key research communities, and alignment with policy and industrial practices will be essential to bridge the existing gap. Such efforts may ultimately enable the development of a more coherent and operational understanding of “sustainable material”, where both qualitative interpretation and quantitative considerations can contribute to transforming the current conceptual ambiguity into a more structured and actionable map for the field.

Overall, the present work serves as a conceptual step by highlighting the gap that currently limits consistent interpretation and communication of sustainability in materials science, while clarifying the limitations that must be addressed before more structured and context-dependent classification approaches can be developed.

## Figures and Tables

**Table 1:** **Conceptual overview of commonly used terms related to sustainable materials, highlighting their scope, limitations, contextual interpretation, and representative examples across different material classes**. It illustrates that widely used terms, i.e., bio-based, recyclable, or biodegradable, each capture specific aspects of material behavior, but do not alone define “sustainable material”, and should be applied differently depending on context and application.

Term	Primary Focus	What It Captures	What It Does Not Capture	Common Misinterpretation	Representative Material Examples
**Eco-friendly**	General perception	Broad environmental benefit (often qualitative)	Measurable criteria, lifecycle impacts	Used without a scientific definition	Consumer packaging, coatings, and household plastics
**Green**	General claim	Reduced impact in selected aspects	Full lifecycle performance, trade-offs	Treated as equivalent to sustainable	Green solvents, green polymers
**Renewable**	Resource origin	Regenerating feedstock over time	Processing burden, land use, performance	Assumed inherently sustainable	Wood, bamboo, cellulose
**Bio-based**	Resource origin	Biological or biomass-derived feedstocks	Energy use, emissions, embodied carbon, end-of-life behavior	Confused with biodegradable or sustainable	* PLA, * Bio-PE, starch-based polymers, especially in packaging and lightweight applications
**Recycled**	Material input	Use of previously processed material	Quality degradation, energy for reprocessing, and associated embodied impacts	Assumed zero environmental impact	Recycled aluminum, recycled * PET, * GTR, particularly in packaging, automotive, and secondary manufacturing applications
**Recyclable**	Potential end-of-life	Ability to be reprocessed in principle	Actual collection, sorting, infrastructure, energy demand of recycling systems	Confused with recycled in practice	* PET bottles, glass, aluminum, widely used in the packaging and construction sectors
**Biodegradable**	End-of-life behavior	Ability to degrade under certain conditions	Required conditions, degradation time, possible emissions during degradation	Assumed universal degradation	* PLA, starch blends, * PHA, often considered for packaging and controlled disposal applications
**Compostable**	Controlled degradation	Degradation under industrial composting conditions	Availability of composting systems	Confused with biodegradable in nature	Compostable packaging films
**Low-carbon**	Emissions	Reduced CO_2_ emissions in specific stages, often reflected through embodied carbon considerations	Other impacts such as toxicity, resource use, broader lifecycle trade-offs	Equated with overall sustainability	Low-carbon steel, green cement, often produced using alternative energy sources or decarbonized processes
**Circular**	System concept	Compatibility with closed-loop systems	Implementation feasibility, system losses, infrastructure dependence	Assumed automatically circular	Metals, paper, some polymers, especially within organized reuse and recycling systems
**Sustainable**	Multidimensional concept	Combined performance across multiple aspects	Lacks a structured definition and requires context	Used as an umbrella or a vague label	All material classes (context-dependent and application-specific)

* **Abbreviations:** Polyethylene terephthalate (PET); Ground Tire Rubber (GTR); Polylactic acid (PLA); Bio-polyethylene (Bio-PE); Polyhydroxyalkanoates (PHA).

## Data Availability

No new data were created or analyzed in this study. Data sharing is not applicable to this article.

## References

[1] Kar S., Sanderson H., Roy K., Benfenati E., Leszczynski J. (2022). Green Chemistry in the Synthesis of Pharmaceuticals. Chem. Rev..

[2] Jones G.R., Wang H.S., Parkatzidis K., Whitfield R., Truong N.P., Anastasaki A. (2023). Reversed Controlled Polymerization (RCP): Depolymerization from Well-Defined Polymers to Monomers. J. Am. Chem. Soc..

[3] Liu C., Pham M.-S. (2024). Spatially Programmable Architected Materials Inspired by the Metallurgical Phase Engineering. Adv. Mater..

[4] Juraij K., Sagitha P., Sujith A., Chinglenthoiba C. (2025). Electrospun nanofibrous membranes based sorbents for oil/water separation: Blends, composites, functionalized, and smart membranes—A mini review. J. Mater. Res. Technol..

[5] Mauro J.C., Tandia A., Vargheese K.D., Mauro Y.Z., Smedskjær M.M. (2016). Accelerating the design of functional glasses through modeling. Chem. Mater..

[6] Curtarolo S., Hart G.L.W., Buongiorno Nardelli M., Mingo N., Sanvito S., Levy O. (2013). The high-throughput highway to computational materials design. Nat. Mater..

[7] Bandyopadhyay A., Heer B. (2018). Additive manufacturing of multi-material structures. Mater. Sci. Eng. R Rep..

[8] Dumée L.F. (2022). Circular Materials and Circular Design—Review on Challenges Towards Sustainable Manufacturing and Recycling. Circ. Econ. Sustain..

[9] Allwood J.M. (2016). Sustainable materials. Nat. Rev. Mater..

[10] Glavič P., Lukman R. (2007). Review of sustainability terms and their definitions. J. Clean. Prod..

[11] Glavič P. (2021). Evolution and Current Challenges of Sustainable Consumption and Production. Sustainability.

[12] Oh J.Y., Lee Y., Lee T.-W. (2024). Skin-Mountable Functional Electronic Materials for Bio-Integrated Devices. Adv. Healthc. Mater..

[13] Huang G., Huang F., Dong W. (2024). Machine learning in energy storage material discovery and performance prediction. Chem. Eng. J..

[14] Jia X., Li Y., Shen B., Zheng W. (2022). Evaluation, fabrication and dynamic performance regulation of green EMI-shielding materials with low reflectivity: A review. Compos. Part B Eng..

[15] Andreu A., Lee H., Kang J., Yoon Y.-J. (2024). Self-Healing Materials for 3D Printing. Adv. Funct. Mater..

[16] Yan L., Xu H. (2025). Lightweight composite materials in automotive engineering: State-of-the-art and future trends. Alex. Eng. J..

[17] Jiao P., Mueller J., Raney J.R., Zheng X., Alavi A.H. (2023). Mechanical metamaterials and beyond. Nat. Commun..

[18] Ljungberg L.Y. (2007). Materials selection and design for development of sustainable products. Mater. Des..

[19] Sugrañez R., Cruz-Yusta M., Mármol I., Martín F., Morales J., Sánchez L. (2012). Use of industrial waste for the manufacturing of sustainable building materials. ChemSusChem.

[20] Olivetti E.A., Cullen J.M. (2018). Toward a sustainable materials system. Science.

[21] Glavič P., Levičnik H., Szilagyi A., Zugasti I., Schönfelder T., Rosicki M., Ruzicka P., Hajná V. (2025). Smart Education for Corporate Sustainability Reporting. Standards.

[22] Glavič P. (2026). Navigating Unserved Areas: A Comprehensive Review of Medical Deserts. Standards.

[23] Glavič P., Novak Pintarič Z., Levičnik H., Dragojlović V., Bogataj M. (2023). Transitioning towards Net-Zero Emissions in Chemical and Process Industries: A Holistic Perspective. Processes.

[24] Ben-Eli M.U. (2018). Sustainability: Definition and Five Core Principles, a Systems Perspective. Sustain. Sci..

[25] Rahim A.A.A., Musa S.N., Ramesh S., Lim M.K. (2020). A systematic review on material selection methods. Proc. Inst. Mech. Eng. Part L J. Mater. Des. Appl..

[26] Kim E., Huang K., Saunders A., McCall K.M., Liu H., Cole J.M., Olivetti E.A. (2020). Machine-learned metrics for predicting the likelihood of success in materials discovery. npj Comput. Mater..

[27] Veleva V., Hart M., Greiner T., Crumbley C. (2001). Indicators of sustainable production. J. Clean. Prod..

[28] Valsami-Jones E., Cassee F.R., Falk A. (2022). From small to clever: What does the future hold for the safety and sustainability of advanced materials?. Mater. Today Sustain..

[29] Greene J.P. (2022). Sustainable Plastics: Environmental Assessments of Biobased, Biodegradable, and Recycled Plastics.

[30] McDonough W., Braungart M. (2002). Cradle to Cradle: Remaking the Way We Make Things.

[31] Pawanr S., Gupta K. (2024). A Review on Recent Advances in the Energy Efficiency of Machining Processes for Sustainability. Energies.

[32] Čuček L., Klemeš J.J., Kravanja Z. (2012). A Review of Footprint analysis tools for monitoring impacts on sustainability. J. Clean. Prod..

[33] Trucillo P., Erto A. (2023). Sustainability Indicators for Materials and Processes. Sustainability.

[34] Zhang Z., Zheng Y., Qian L., Luo D., Dou H., Wen G., Yu A., Chen Z. (2022). Emerging Trends in Sustainable CO_2_-Management Materials. Adv. Mater..

[35] Moshood T.D., Rotimi J.O.B., Wajiha S. (2023). Sustainability principles in infrastructure project delivery: Establishing the broader implementation strategies for decision-making. Constr. Innov..

[36] Arvidsson R., Svanström M., Sandén B.A., Thonemann N., Steubing B.R.P., Cucurachi S. (2024). Terminology for future-oriented life cycle assessment: Review and recommendations. Int. J. Life Cycle Assess..

[37] Susur E., Karakaya E. (2021). A reflexive perspective for sustainability assumptions in transition studies. Environ. Innov. Soc. Transit..

[38] Chong Y.T., Teo K.M., Tang L.C. (2016). A lifecycle-based sustainability indicator framework for waste-to-energy systems and a proposed metric of sustainability. Renew. Sustain. Energy Rev..

[39] Shi J., Duan K., Wu G., Si H., Zhang R. (2022). Sustainability at the Community Level: A Bibliometric Journey around a Set of Sustainability-related Terms. Sustain. Dev..

[40] Tecchio P., McAlister C., Mathieux F., Ardente F. (2017). In search of standards to support circularity in product policies: A systematic approach. J. Clean. Prod..

[41] Glavič P. (2022). Updated Principles of Sustainable Engineering. Processes.

[42] Krajnc D., Glavič P. (2003). Indicators of sustainable production. Clean Technol. Environ. Policy.

[43] Arora N.K., Mishra I. (2019). United Nations Sustainable Development Goals 2030 and environmental sustainability: Race against time. Environ. Sustain..

[44] Ekert R., Kovalevska O. (2021). Sustainability in the European Union: Analyzing the Discourse of the European Green Deal. J. Risk Financ. Manag..

[45] Amran A., Lee S.P., Devi S.S. (2014). The Influence of Governance Structure and Strategic Corporate Social Responsibility Toward Sustainability Reporting Quality. Bus. Strategy Environ..

[46] Stawicka E. (2025). Environmental issues and digital transformation in sustainability reporting in enterprises in Poland. Econ. Envir..

[47] Sharma R. (2025). Unveiling the effects of the Corporate Sustainability Reporting Directive (CSRD) on company sustainability reporting practices: A case of German companies. Sustain. Account. Manag. Policy J..

[48] Leal Filho W., Wall T., Williams K., Pimenta Dinis M.A., Fernandez Martin R.M., Mazhar M., Gatto A. (2025). European sustainability reporting standards: An assessment of requirements and preparedness of EU companies. J. Environ. Manag..

[49] Nial N., Parashar P. (2024). A comparative study on sustainability standards with specific reference to GRI standards and BRSR framework. Int. J. Qual. Reliab. Manag..

